# Mechanisms of *Clostridioides difficile* glucosyltransferase toxins and their roles in pathology: insights and emerging therapeutic strategies

**DOI:** 10.3389/fcimb.2025.1641564

**Published:** 2025-10-13

**Authors:** Xin Wen, Xue Liu, Kai Wan, Hong Liu, Cheng Zhang, Xi Zhang, Qin Wen

**Affiliations:** Medical Center of Hematology, Xinqiao Hospital of Army Medical University; Chongqing Key Laboratory of Hematology and Microenvironment, State Key Laboratory of Trauma and Chemical Poisoning, Army Medical University, Chongqing, China

**Keywords:** *Clostridioides difficile infection*, glucosyltransferase toxins, TcdA and TcdB, pathogenicity, therapeutic strategies

## Abstract

*Clostridioides difficile* infection (CDI) is a significant cause of antibiotic-associated diarrhea and pseudomembranous colitis, manifesting as mild diarrhea, fulminant colitis, and even death. It is typically recognized as a healthcare-associated infection. Glucosyltransferase toxin A (TcdA) and toxin B (TcdB) are two major factors responsible for the pathogenicity of *Clostridioides difficile* (*C. difficile*). They bind to cell surface receptors and enter the cytoplasm via pH-dependent pore formation, causing cell death by inactivating GTPase. This review elucidates the pathogenic mechanisms of *C. difficile* glucosyltransferase toxins and discusses the interactions between the two toxins and host cells. It also summarizes current progresses in CDI therapies, providing a comprehensive understanding of the disease and laying the foundation for developing novel therapies and management strategies.

## 1 Introduction

*Clostridioides difficile* (*C. difficile*) is an obligate anaerobic, spore forming, Gram-positive bacillus, which was first isolated from the stool samples of healthy infants in 1935 and initially recognized as a kind of normal gut flora (Hall, 1935; Kelly et al., 1994b). It was not classified as an enteric pathogen until Bartlett and colleagues isolated toxin-producing *C. difficile* from the feces of patients with antibiotic-associated pseudomembranous colitis in 1978 (Bartlett et al., 1978). In an oxygen-rich environment, *C. difficile* forms spores to resist tough external conditions such as dryness, high temperatures, extreme pH levels, and even lethal effects of various chemicals and disinfectants (Paredes-Sabja et al., 2014). Due to the potent spreading capacity, the spores widely exist in medical environment and result in an inundate spread of *C. difficile* in the health system (Martin et al., 2016). The clinical manifestations of *Clostridioides difficile* infection (CDI) exhibit varied degrees of severity. These range from the mild forms such as asymptomatic colonization and mild diarrhea, to the severe conditions including pseudomembranous colitis, toxic megacolon, bowel perforation, and even death (Kociolek and Gerding, 2016). The occurrence of CDI is correlated with several high-risk factors, such as long-term use of antibiotics, weakened immune systems, severe underlying conditions, invasive procedures such as surgery, prolonged hospitalization, and advanced age (Bartlett, 2002; Leffler and Lamont, 2015; Wang et al., 2024).

**Figure 1:**
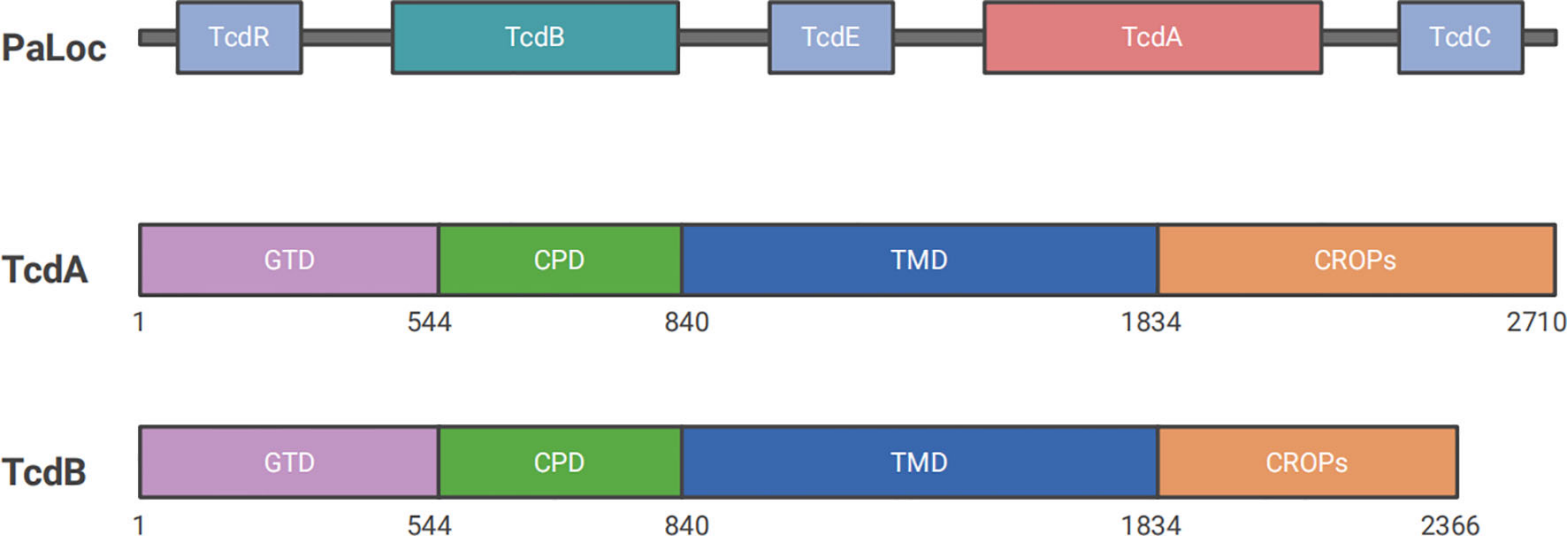
Structures of Clostridioides difficile PaLoc. PaLoc primarily encodes five genes: *tcdR*, *tcdB*, *tcdE*, *tcdA*, and *tcdC*. *TcdA* and *tcdB* encode two of the most important toxin proteins, TcdA and TcdB, which are responsible for CDI pathogenesis. Both toxins consist of four domains: CROP, which binds to target cell surface receptors; TMD, which is involved in the delivery process; CPD, a self-hydrolytic domain that cleaves and releases GTD into the cytoplasm to exert enzymatic functions; and GTD, which inactivates small GTPases to induce cell death.

**Figure 2:**
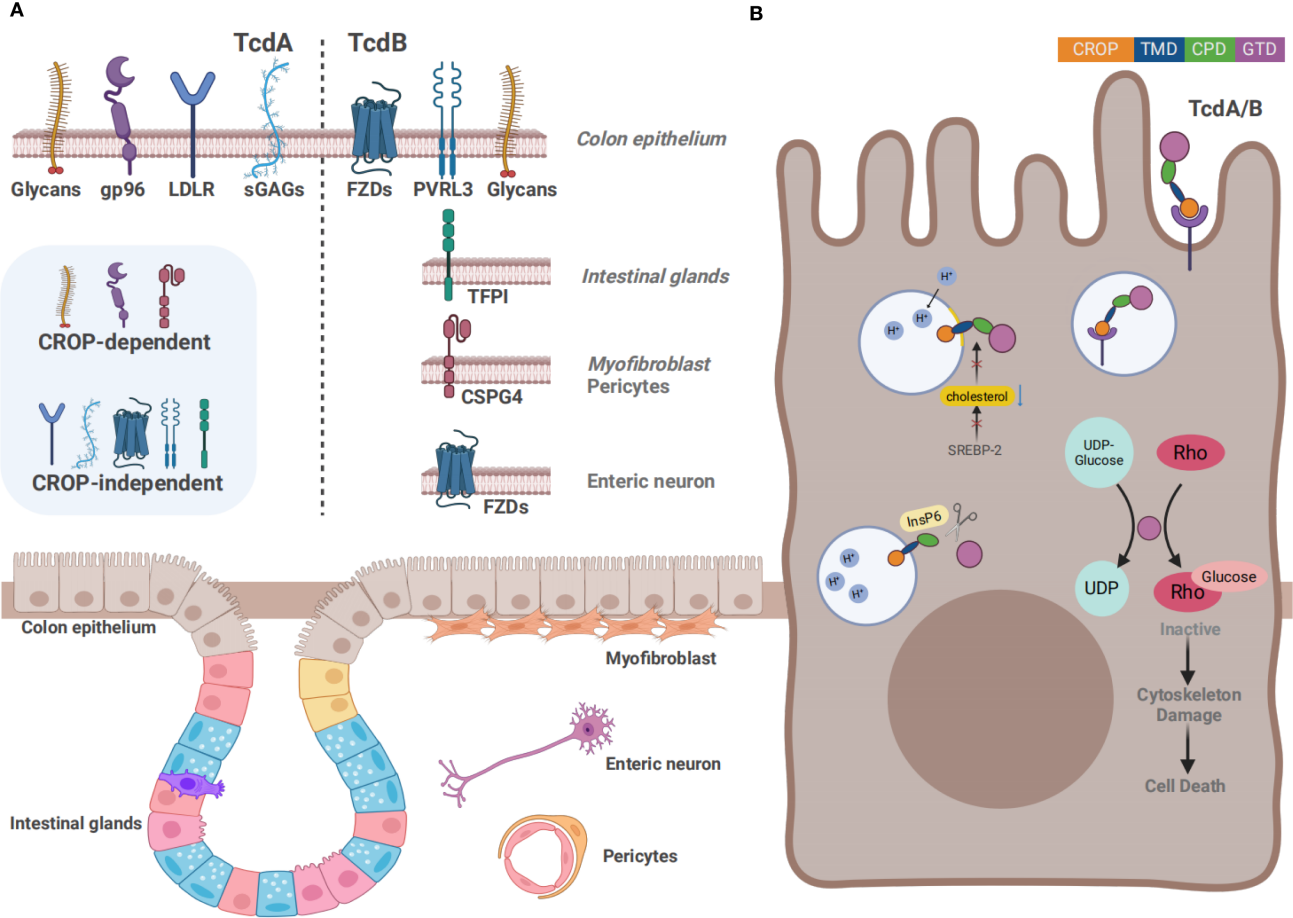
Overview of the action mechanism of TcdA and TcdB **(A)** The distinct cell surface receptors of TcdA and TcdB. Gp96, sGAGs, LDLR, and glycans such as Lewis X/Y/I have been confirmed as the receptors for TcdA, whereas CSPG4, PVRL3, FZDs, and TFPI are key receptors for TcdB. In addition to the CROP-dependent receptors, several receptors bind to the toxins independently of the CROP domain. Except for IECs, receptors are expressed on several other cell types, including intestinal glands, myofibroblasts, pericytes, and enteric neurons. **(B)** The action mechanism of TcdA and TcdB. Firstly, the toxins bind to the cell surface receptors through the CROP domain or other structure, followed by the internalization of toxins in acidic endosomes formed by endocytosis. Acidic endosomes subsequently trigger the pore formation and transport the CPD and GTD into the cytosol. Next, autocatalytic cleavage of the toxins is processed in the presence of InsP6, by which GTD is released into the cytosol. Rho GTPases are inactivated by transferring UDP-glucose to them, ultimately resulting in the induction of cytoskeletal damage.

The pathogenesis of CDI is driven by two types of toxins: large clostridial toxins (LCTs) TcdA and TcdB, and *C. difficile* transferases (CDT). TcdA and TcdB are considered the major virulence factors, each consisting of four functional domains: a glucosyltransferase domain (GTD), a cysteine proteinase domain (CPD), a transmembrane domain (TMD), and a C-terminal repetitive oligopeptide domain (CROP) (**Figure 1**) (Chandrasekaran and Lacy, 2017). TcdA and TcdB are internalized via receptor-mediated endocytosis. Glycoprotein 96 (gp96), sulfated glycosaminoglycans (sGAGs), and low-density lipoprotein receptor (LDLR) have been confirmed as the receptors for TcdA (Na et al., 2008; Tao et al., 2019; Schottelndreier et al., 2020), whereas chondroitin sulfate proteoglycan 4 (CSPG4), poliovirus receptor-like 3 (PVRL3), frizzled family (FZDs), and tissue factor pathway inhibitor (TFPI) are key cellular factors that mediate the binding and endocytosis of TcdB (**Figure 2A**) (Tao et al., 2016; Chen et al., 2021; Tian et al., 2022; Childress et al., 2023). Upon entering the cell, GTD is released into the cytoplasm through a pH-dependent autocleavage process. GTD inactivates GTPases through its glucosyltransferase (GT) activity, leading to disruption of the actin cytoskeleton and ultimately inducing cell death (**Figure 2B**) (Chandrasekaran and Lacy, 2017). Although both toxins ultimately result in cell death, TcdA is believed to induce apoptosis in a GT-dependent manner. In contrast, TcdB exhibits dose-dependent cytotoxicity, with apoptosis occurring at low doses in a GT-dependent manner and necrotic cell death at high doses in a GT-independent manner (Peritore-Galve et al., 2022).

Although treatments for CDI vary worldwide, antibiotics continue to be the first-line treatment option. However, due to its high mortality and recurrence rates, the prevention and treatment of CDI remain a great challenge in the field of healthcare (Maroo and Lamont, 2006; Surawicz, 2009). Except for antibiotics, scientists have been focusing on developing other therapeutic strategies, including monoclonal antibodies (mAbs), vaccines, gut microbiota restoration, and natural small molecular products. For example, fecal microbiota transplantation (FMT) was proposed as a treatment for recurrent *Clostridioides difficile* infection (rCDI) in 2013 (van Nood et al., 2013). Evidence from a phase III clinical trial indicated that the anti-TcdB mAb bezlotoxumab significantly reduced the recurrence rate of CDI in 2017 (Wilcox et al., 2017). In the same year, fidaxomicin replaced metronidazole as the first-line treatment for initial CDI episode (McDonald et al., 2018). With the deepening of the research on CDI therapies, the development of bioactive molecules and vaccines continues to provide new aspects for the disease prevention and treatment (Kordus et al., 2021; Bratkovic et al., 2024; Remich et al., 2024). To date, CDI remains an extremely complex issue that has garnered significant interest among researchers. This review focuses on elaborating the epidemiology of CDI, the mechanisms of toxin action, the toxin–host interaction pathways and recent advances in therapies. It aims to provide a comprehensive understanding of the disease and sketch a general view for developing novel therapies and management strategies for CDI.

## 2 Epidemiology and medical burden of *Clostridioides difficile* infection

CDI has become a concerning challenge worldwide, with rising trends in incidence and mortality rates. The epidemiological situation in medical institutions is even worse (Di Bella et al., 2024). Statistics indicate that in hospitals in North America and Europe, the annual incidence of CDI has reached approximately 4~10/1,000 cases of hospitalized patients (Rupnik et al., 2009). Compared to other healthcare-associated infections, CDI has a higher mortality rate. According to statistics from the Centers for Disease Control and Prevention, approximately 453,000 cases of CDI occur annually in the United States. These cases result in about 29,000 deaths and impose $1.5 billion in healthcare costs (Lessa et al., 2015). Moreover, CDI has a relatively high recurrence rate of about 20% to 30% (Wingen-Heimann et al., 2023). Numerous clinical trials conducted in Germany, France, Japan, and other countries have demonstrated that patients with rCDI experience longer hospital stays and incur higher medical costs than those with initial CDI, placing a heavy burden on the healthcare system (Heimann et al., 2015; Dinh et al., 2019; Kimura et al., 2020). Therefore, early diagnosis and treatment of CDI patients can reduce the risks of complications, recurrence, and infection-associated death.

The epidemiological characteristics of CDI vary by region and time, which have been evolving during the past three decades. It was initially prevalent in Western countries, then a sharp rise in CDI incidence and mortality rate occurred from 1991 to 2003, followed by its spread worldwide (Pepin, 2004). Later, McDonald et al. in the United States identified a new strain with greater virulence, namely NAP1/RT027. This strain is resistant to fluoroquinolones and rapidly spreads to Europe before prevailing worldwide, severely affecting human health and healthcare costs (McDonald et al., 2005; Arvand et al., 2014; Zhou et al., 2019). From 1999 to 2004, statistics showed a fourfold increase of CDI-related mortality in the United States (Redelings et al., 2007). Owing to the restrictions on the use of fluoroquinolone antibiotics, healthcare-associated events have decreased more or less since 2011. However, the prevalence of CDI remains widespread (Dingle et al., 2017). In recent years, another *C. difficile* strain, RT017, expressing TcdB only and exhibiting relatively strong virulence and spreading capacity, is prevailing rapidly from Asia to the whole world and attracting significant attention (Cairns et al., 2015; Imwattana et al., 2019). This aligns with our previous research on the epidemic and nosocomial transmission of *C. difficile* in China (Wen et al., 2022). The severity of this public health issue cannot be ignored, and it is of great significance to enhance the supervision over CDI and devote to clarifying its pathogenic mechanisms, so as to develop more effective clinical preventive and therapeutic strategies.

## 3 Overview of *Clostridioides difficile* toxins

The pathogenesis of *C. difficile* is mediated by two pathogenic islands: the pathogenicity locus (PaLoc) and the binary toxin encoding locus (CdtLoc). PaLoc is the primary pathogenic locus present in all toxic isolates, while CdtLoc is present only in a minority of PCR ribotypes (Awad et al., 2014).

TcdA and TcdB, two macromolecular proteins with molecular weights of 308 kDa and 270 kDa respectively, are encoded by specific genes on the PaLoc with a size of 19.6 kb. Another three genes are located on the PaLoc, including *tcdR*, *tcdE*, and *tcdC*, which primarily function in regulating the expression of the toxins (Dingle et al., 2014; Chandrasekaran and Lacy, 2017). TcdA and TcdB are two principal toxins responsible for CDI and belong to the LCT family (Orrell and Melnyk, 2021). Both of them comprise four functional domains: GTD, CPD, TMD, and CROP. Each domain plays an important role during the activation process of biological virulence. Firstly, the CROP domain binds to cell surface receptors (such as glycoproteins and glycolipids) to mediate cellular internalization by initiating endocytosis to form vesicles. Secondly, the conformational change of TMD under acidic environment facilitates the toxins to cross the cell membrane by the pore formation. Then, catalysed by inositol hexaphosphate (InsP6), CPD performs autocleavage by hydrolyzing specific protein substrates to release GTD into the cytoplasm. Finally, GTD, as a glycosylation enzyme, inactivates small GTPases of the Rho/Ras subfamily by adding glucose molecules in the cytoplasm (Jank and Aktories, 2008; Abt et al., 2016). Signaling proteins concluded in Rho/Ras subfamily regulate actin-dependent processes, such as cell migration, phagocytosis, and cell contraction. They also participate in various signaling pathways that control gene expression, cell cycle, and apoptosis (Jank et al., 2007a; Alam and Madan, 2024). As the first discovered toxin of *C. difficile*, TcdA has received extensive attention in various studies. TcdA was formerly known as an enterotoxin because it possessed intestinal cell adhesion properties. It mainly caused intestinal inflammation, exudation, and mucosal damage by disrupting intestinal barrier function (Triadafilopoulos et al., 1987; Smits et al., 2016). Evidence from previous studies has shown that TcdA is more effective in promoting secretion, causing mucosal damage, and initiating inflammation compared to TcdB (Triadafilopoulos et al., 1987). In contrast, TcdB was known as a cytotoxin that primarily functioned by disrupting the cytoskeleton and enhancing cell permeability, which led to cell rounding and death (Mileto et al., 2020). However, the specific roles and mechanisms of both toxins remain controversial due to conflicting evidence in the literature. An early study found that TcdA was shown to cause clinical symptoms independently, whereas TcdB toxicity depended on the presence of TcdA or pre-existing damage to the intestinal mucosa in animal models (Lyerly et al., 1985). Following, a report published in Nature in 2009 proposed that TcdB played a crucial role in pathogenesis, while strains expressing only TcdA were non-pathogenic *in vitro* and in a hamster disease model (Lyras et al., 2009). Shortly after, another study has claimed a different conclusion: both TcdA and TcdB are toxic (Kuehne et al., 2010). Researchers have speculated that the discrepancies arose from SNPs in toxin sequences, which is confirmed by the studies afterwards (Lanis et al., 2010; Rupnik and Janezic, 2015; Knight et al., 2015). The homology of amino acid sequences between TcdA and TcdB is 63% (von Eichel-Streiber et al., 1992). A study suggested that TcdB had a 100 to 1,000-fold higher efficacy than TcdA at the cellular level (Riegler et al., 1995). Building on this, another study using three different animal models demonstrated that TcdB played a more significant role in CDI, potentially leading to multiple organ dysfunction syndrome (Carter et al., 2015). Additionally, recent studies have shown that TcdA and TcdB target different receptors during the exertion of their toxic effects, leading to varying degrees of host immune and inflammatory responses (Liu et al., 2021a; Li and Saavedra, 2025). It is suggested that TcdB may play a more critical role in inducing cell death and promoting disease progression.

The CdtLoc, which is 6.2 kb in size, comprises three genes: *cdtA*, *cdtB*, and *cdtR*, among which *cdtA* and *cdtB* encode CDT to enhance the adhesion of *C. difficile* to target cells, while *cdtR* plays a regulatory role (Aktories et al., 2018). CDT, a binary ADP-ribosylation toxin, is detected in 5-30% of clinical *C. difficile* isolates, which tend to have higher virulence, such as RT027 and RT078 (Gerding et al., 2014). It consists of two parts: chain A and B, where chain A shows enzymatic activity and is responsible for ADP-ribosylation, and chain B is responsible for binding to host cell surface receptors, thereby mediating toxin entry. Chain A is activated after the toxin entry, which is able to transfer ADP-ribose groups to actin in the host cell, leading to changes in actin polymerization. The toxins affect cytoskeleton stability by modifying actin, thereby interfering with cell division and migration (Aktories et al., 2017).

These toxins work together to destroy the colon epithelium, causing the symptomatic manifestations of CDI, such as fluid secretion, inflammation, and tissue damage (Lyerly et al., 1988; Voth and Ballard, 2005; Kordus et al., 2021; Skinner et al., 2021; Pourliotopoulou et al., 2024). Although the enzymatic functions of the toxins have been majorly determined, the mechanism of toxin-host cell interaction remains unclear. Below, we will discuss the progress on the GT toxins, TcdA and TcdB.

### 3.1 The cellular receptors of TcdA and TcdB

The CROP domain, also known as the receptor-binding domain, is recognized as a necessary component for the interaction between toxins and host cell, which can mediate the initiation of toxin internalization. The CROP region of TcdA and TcdB binds to distinct surface receptors (**Figure 2A**) (Dingle et al., 2008). TcdA has been reported to bind to several protein receptor candidates, such as gp96, sGAGs, sucrase-isomaltase (SI), LDLR, Galα1-3Galβ1-4GlcNAc, and Lewis X/Y/I glycans. Nevertheless, there remain inconsistencies in related experimental data. Early studies indicated that TcdA bound to the trisaccharide Galα1-3Galβ1-4GlcNAc *in vitro*. However, this trisaccharide is not naturally expressed on human cells (Krivan et al., 1986; Clark et al., 1987). Another study indicated Lewis X/Y/I glycans as potential receptors for TcdA, which indeed exist on the human intestinal epithelial cells (IECs) (Tucker and Wilkins, 1991). TcdA can also bind to SI, a glycoprotein located on the brush-like edge of the rabbit small intestine (Pothoulakis et al., 1996). However, no such reports have been documented in human IECs. Gp96, a member of the heat shock protein family, is expressed on the endoplasmic reticulum as well as human colonocytes. Gp96 is regarded as one of the cell surface receptors for TcdA. However, gp96-deficient cells are only partially resistant to TcdA, suggesting that TcdA may also bind to other receptors (Na et al., 2008). Meanwhile, another study has indicated that gp96 is a binding receptor, whereas low-density lipoprotein receptor-associated protein 1 (LRP1) acts as an endocytic receptor for TcdA (Schottelndreier et al., 2020). Receptors LDLR and sGAGs are ubiquitously expressed on different mammalian cell surfaces (Jinno and Park, 2015). A recent study identified sGAGs and LDLR as CROP-independent host factors through genome-wide CRISPR-cas9 mediated screen technology, since both of them can mediate the binding and entry of the truncated TcdA which lacks the CROP domain (Tao et al., 2019). Subsequently, another *in vitro* study revealed that blocking the sGAGs could effectively inhibit the endocytosis of TcdA, further confirming that sGAGs played a crucial role in the endocytosis of TcdA (Zhang et al., 2025). Additionally, recent studies have found that the CROP domain exhibits a pH-dependent dynamic characteristic, which likely plays a crucial role in the cytotoxicity of TcdA (Chen et al., 2022a; Aminzadeh et al., 2022).

TcdB has been reported to bind to CSPG4, PVRL3, FZDs, TFPI, and a variety of glycans. CSPG4 is also known as neuron-glial antigen 2, which is highly expressed in sub-epithelial myofibroblast cells within the colonic tissues instead of the colonic epithelium (Tamburini et al., 2019). It was firstly confirmed as a CROP-dependent TcdB receptor through shRNA-mediated knock-down screen in 2014 (Yuan et al., 2015). There is a direct interaction between the N-terminus of CSPG4 and the C-terminus of TcdB, which can be promoted by extracellular Ca^2+^. The soluble peptide in the toxin-binding domain of CSPG4 can protect cells from TcdB (Yuan et al., 2015; Doyle et al., 2024). Meanwhile, another study showed that the cytotoxicity of TcdB was reduced when the expression of CSPG4 was down-regulated by inhibiting the Hippo signaling pathway (Larabee et al., 2023). However, TcdB was found to reduce IL-8 expression in CSPG4-knockout mice, but the mortality of CSPG4-knockout mice was not significantly different from that of the wild type mice, indicating that there may exist other receptors for TcdB (Yuan et al., 2015). Therefore, researchers have proposed the dual-receptor model for TcdB endocytosis, and the presence of alternative receptors was further confirmed by bezlotoxumab. It is an anti-TcdB antibody approved by the US Food and Drug Administration (FDA). The inhibition of TcdB binding to CSPG4 by the allosteric mechanism of bezlotoxumab did not show significant neutralization efficacy against numerous TcdB variants from prevalent hypervirulent strains (Chen et al., 2021). In the same year, another novel TcdB receptor, LRP1, was discovered by CRISPR-Cas9 screening in CSPG4-deficient HeLa cells by Shengjie Guo and his colleagues (Guo et al., 2022). However, previous research has indicated that LRP1 is not the endocytic receptor for TcdB in fibroblasts, suggesting that toxin receptors may exhibit cell type specificity (Schottelndreier et al., 2020). PVRL3, also known as Nectin-3, was identified as a cellular factor by a genetrap insertional mutagenesis screen, which was necessary for TcdB-mediated necrotic cell death. Additionally, it binds to TcdB independently of the CROP domain (LaFrance et al., 2015; Sakisaka and Takai, 2004). PVRL3 is highly expressed on the epithelial surface of the human colon. A study has revealed the unexpected localization of PVRL3 on the brush border of colonic epithelial cells by immunofluorescence microscopy, which is different from the localization of CSPG4 at epithelial cell junctions (Childress et al., 2023). In 2016, members of the Wnt receptor FZDs were identified as TcdB receptors by CRISPR/Cas9-mediated genome-wide screening by Liang Tao and his colleagues (Tao et al., 2016). Unlike CSPG4, FZDs are CROP-independent receptors. The FZDs family is a group of 7-pass transmembrane proteins. FZDs possess a unique extracellular domain termed as the cysteine-rich domain (CRD), which serves as the binding site of Wnt (Chen et al., 2018). It consists of 10 human genes (FZD1-10), among which FZD1/2/7 share sequence similarity of approximate 98% and are confirmed to facilitate TcdB entry into HeLa cells (MacDonald and He, 2012). Since both TcdB and Wnt bind to the FZD-CRD, it is theoretically feasible that the interaction between TcdB and FZDs may directly contribute to the disruption of the colon epithelium by blocking Wnt signaling (Chen et al., 2019). Another study has indicated that TcdB from epidemic NAP1/RT027 strains induced the dysfunctional stem cell state in both mice and human colonic organoids without binding to FZD1/2/7, instead it maintained the ability to interact with CSPG4 and Nectin-3 (Mileto et al., 2020). This suggested that different TcdB variants may adapt to various receptors. Recently, a number of studies have indicated that TcdB variants entered host cell by binding to distinct receptors, although they shared similar substrate profiles and cytotoxicity (Lopez-Urena et al., 2019; Henkel et al., 2020). In 2021, Liang Tao’s team has proposed that TcdB variants presented highly diversified receptor preferences: TcdB1 binds to two known receptors CSPG4 and FZDs, TcdB2 selectively binds to CSPG4, TcdB3 tends to interact with FZDs, and TcdB4 exerts toxic effects in a CSPG4/FZDs-independent manner (Pan et al., 2021). TFPI is highly expressed in the intestinal glands. Moreover, a recent study has identified TFPI as a colonic crypt receptor for TcdB from clade2 strain, a clinically prevalent and highly virulent strain. The severity of the toxic effects of clade2 strain may be related to the specific receptor of this TcdB variant (Tian et al., 2022; Luo et al., 2022).

With the development of the genome-wide CRISPR-Cas9 technology, researchers have identified a series of molecules as host receptors for TcdA and TcdB. While TcdA receptors remain conserved, TcdB subtypes are highly diversified in receptor specificity, translocation ability, inflammatory responses, and pathological outcomes. Several studies have shown that the family of *Clostridioides* GT toxins enter cells by binding to multiple receptors, with more than one host target (Manse and Baldwin, 2015; Lambert and Baldwin, 2016; Pan et al., 2021). With the proposal of the dual-receptor model, receptor binding sites for TcdA and TcdB are apparently not limited to the CROP domain. Recently, studies on toxin receptor revealed that TcdA and TcdB lacking the CROP domain could still interfere with host cell function, reflecting the presence of additional receptor binding regions (Schorch et al., 2014; Yuan et al., 2015; LaFrance et al., 2015; Tao et al., 2016; Doyle et al., 2024). Previous research has shown that TcdA and TcdB adopted distinct endocytic pathways. TcdB enters the cells via the clathrin-dependent endocytic pathway (Papatheodorou et al., 2019), while the internalization of TcdA is accomplished by a clathrin- and caveolae-independent mechanism mediated by PACSIN2 and dynamin (Chandrasekaran et al., 2016). However, the pathway by which toxins enter cells remains to be further clarified.

### 3.2 Pore-formation, translocation and autoprocessing

The TMD, also known as the delivery domain, plays the key role in forming pores to mediate the translocation of toxins. Endosomes form after toxins bind to cell surface receptors and are internalized into the cytoplasm through endocytosis. The acidic environment in endosomes is essential for the translocation of the toxins. Decreased pH in endosomes can induce a structural change of TMD, which enhances hydrophobicity and triggers the insertion of the TMD into the membrane, forming a pore-like α-helical structure (Qa’Dan et al., 2000; Orrell et al., 2017, Orrell et al., 2018). Then GTD and CPD translocate into the cytoplasm via the pores (**Figure 2B**). The formation of pores is usually accompanied by the formation of ion channels, which is a phenomenon historically reported for other translocating toxins (Barth et al., 2001). The optimal pH for the hydrophobic transition of TcdB ranges from 4.0 to 5.0, although fluctuations in the optimal pH have been observed among different TcdB subtypes (Lanis et al., 2013). An *in vivo* study has indicated that TcdB from hypervirulent *C. difficile* strains undergoes conformational changes at a higher pH to facilitate itself to translocate into cytoplasm more rapidly during the early stage of endocytosis (Lanis et al., 2010). A structurally related study has defined the minimal pore-forming region of TcdB, which is located at amino acid residues 830 and 990 (Genisyuerek et al., 2011). In an analysis covering over 8,000 *tcdB* genes, the sequence of the TMD was found to be the most evolutionarily conserved, indicating its potential in being an attractive target for broad-spectrum therapeutics (Mansfield et al., 2020). TcdA undergoes conformational changes and forms pores at a low pH level, and the process of pore formation by TcdA is cholesterol-dependent. Meanwhile, similar results were obtained for TcdB, suggesting that pore formation is dependent on the presence of cholesterol (Giesemann et al., 2006a). The sterol regulatory element–binding protein 2 (SREBP-2) pathway plays a crucial role in regulating the cholesterol content in cellular membranes. A recent *in vitro* study has shown that inhibiting the SREBP-2 pathway disrupted the cholesterol-dependent pore formation of TcdB in cell membranes, demonstrating that the SREBP-2 pathway may be a suitable target for antitoxin therapeutics against *C. difficile* toxins (Papatheodorou et al., 2019). Moreover, another *in vitro* study with cultured cells and human intestinal organoids, has found that amiodarone, a clinically common agent for treating cardiac arrhythmia, can inhibit cholesterol biosynthesis and subsequently interfere with the formation of translocation pores, suggesting its potential role in antitoxin therapy (Schumacher et al., 2023).

The CPD, also known as the autoprotease domain, facilitates the autoproteolytic cleavage and releases the GTD into the cytosol. With the pore formation, the GTD and CPD are unfolded and translocated to the host cytoplasm, where their biological activity was recovered through refolding under the assistance of the chaperonin TCP-1 ring complex/chaperonin containing TCP-1 (TRiC/CCT) (Giesemann et al., 2008; Steinemann et al., 2018). In order to initiate autoprocessing, CPD binds to the cellular host factor InsP6. Cytosolic InsP6, a molecule uniquely found within the cytosol of eukaryotic cells, acts as an allosteric activator to initiate the cysteine-protease activity of the CPD (Egerer et al., 2007; Reineke et al., 2007; Pruitt et al., 2009). At neutral pH, autocleavage occurs in the rear of a conserved leucine residue situated between the CPD and GTD, thereby GTD is released into the cytosol (Pfeifer et al., 2003; Rupnik et al., 2005). Though TcdA and TcdB operate by a similar mechanism, TcdB is more susceptible to autocleavage triggered by InsP6 than TcdA (Kreimeyer et al., 2011; Olling et al., 2014; Kordus et al., 2021). TcdB from epidemic NAP1/RT027 strains exhibited enhanced autoprocessing activity *in vitro*, suggesting that the sensitivity towards InsP6-mediated cleavage may be responsible for the varied toxicity of TcdB subtypes (Lanis et al., 2012). Research on crystal structures has claimed that zinc ions were essential for the autoprocessing activity of TcdA and TcdB *in vitro* (Chumbler et al., 2016). Another study proved that S-nitrosylation attenuated the toxicity of TcdA and TcdB in a CDI mouse model, which was further confirmed to be associated with the inhibition of toxin autocleavage (Savidge et al., 2011). Additionally, in mouse and human intestinal model, cysteine protease-mediated autoprocessing has been reported to be involved in the regulation of the proinflammatory activities of TcdA and TcdB (Zhang et al., 2018). To conclude, these studies provide new insights into the development of therapeutics against CDI, and further research on the mechanisms of autoprocessing is required to pave the way for new therapeutic approaches.

### 3.3 Glycosylation of the GTD and its enzymatic activity

Both GT and cysteine proteinase activities are essential for the virulence of *C. difficile*. A study has revealed that the GT activity of TcdB is a crucial factor for its cytotoxicity by screening a single-domain heavy-chain variable region (V_H_H) library, whereas cysteine proteinase activity merely plays a regulatory role in the release of GTD from the entire toxin molecule (Li et al., 2015). The release of GTD into the cytoplasm facilitated the selective transfer of UDP-glucose to the Rho and Ras GTPases, inactivating these GTPases by adding glucose molecules (**Figure 2B**). Rho GTPase serves as a molecular switch that regulates various processes, including the organization of the actin cytoskeleton, cell cycle progression, gene transcription, and the activity of numerous enzymes (Etienne-Manneville and Hall, 2002; Burridge and Wennerberg, 2004; Spiering and Hodgson, 2011; Chen et al., 2015). While Ras GTPase primarily controls cell differentiation and proliferation, angiogenesis, and cell adhesion (Macara et al., 1996; Colicelli, 2004). The inactivation of both proteins ultimately causes cell death. On the contrary, UDP-glucose deficiency renders the cells resistant to these toxins (Chaves-Olarte et al., 1996; Flores-Diaz et al., 1997). The GT activity is crucial for the toxic effects of both TcdA and TcdB. Although their sequences show similarity, TcdA and TcdB inactivate different GTPases in the host (Chaves-Olarte et al., 1997; Zeiser et al., 2013; Chen et al., 2022b). A study focusing on the differences in GT activity and substrate specificity between TcdA and TcdB has yielded an interesting result: TcdA is capable of modifying Rap2A in the Ras family, which is incapable for TcdB (Pruitt et al., 2012). Such difference has indicated that the ability to modify substrates from the Rho and Ras family is a prospective pointcut in understanding the pathogenic mechanisms of TcdA. Another study has proposed that TcdB can glycosylate members of the Rho protein family, including RhoA, Rac1, RhoG, TC10, and Cdc42, whereas TcdA cannot glycosylate RhoG and TC10 (Genth et al., 2008). These findings are significant for understanding the structural and functional distinctions between TcdA and TcdB. Meanwhile, several studies have proposed that toxins from various strains of *C. difficile* exhibit distinct preferences in GTPase substrates, which may contribute to the differential pathogenicity. A biomolecular structure study has further confirmed that TcdB variants selectively modify the structural basis of Rho and Ras GTPases through GTD, potentially causing diverse cytopathic effects in host cells (Liu et al., 2021b). Early *in vitro* studies have found that TcdB glycosylates Thr37 of RhoA, which leads to the degradation of the actin cytoskeleton and thereby causes cell death (Just et al., 1995). Subsequently, researchers have pinpointed Asp270, Arg273, Tyr284, Asn384, and Trp520 as essential amino acid residues for GT activity by alanine scanning techniques (Jank et al., 2007b). Mutations in these positions significantly reduce enzyme activity, indicating that these sites may serve as new therapeutic targets for *C. difficile* toxins.

**Figure 3:**
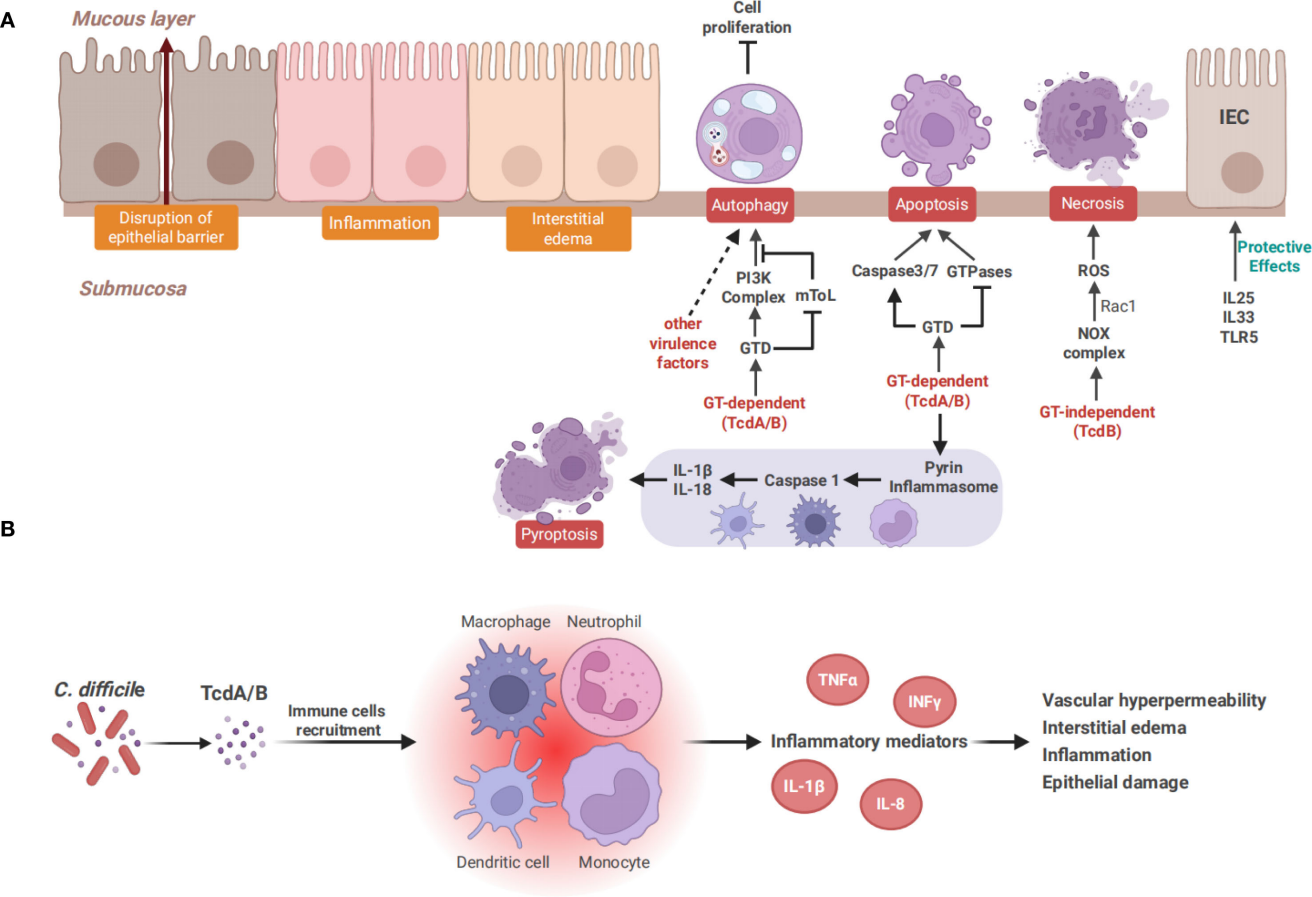
Toxin-mediated intestinal epithelial damage and the inflammatory response. **(A)** TcdA and TcdB cause cell death through distinct mechanisms. TcdA induces apoptosis in a GT-dependent manner, whereas TcdB exhibits dose-dependent cytotoxicity. At a low dose, TcdB induces GT-dependent apoptosis similar to TcdA, while a higher dose of TcdB triggers necrotic cell death. Both TcdA and TcdB can induce pyroptosis in immune cells, including monocytes, macrophages and dendritic cells. Pyroptosis is mediated by pyrin inflammasomes, which activate caspase-1 and subsequently release IL-1β and IL-18. Autophagy is also induced by TcdA and TcdB in a GT-dependent manner and contributes to the inhibition of cell proliferation. It can also be induced by other virulence factors of *C. difficile*, such as surface layer protein **(A, B)** TcdA and TcdB recruit immune cells such as neutrophils, monocytes, and macrophages, subsequently triggering the secretion of inflammatory mediators like TNF-α, IL-1β, and IL-8, leading to increased vascular permeability, interstitial edema, and intestinal epithelial damage, finally causing various forms of cell death.

Repairing the epithelium damaged by CDI and maintaining intestinal integrity are key steps in preventing rCDI. The Wnt/β-catenin pathway is a primary driver for epithelial cell proliferation in colonic crypts. An *in vitro* study has shown that TcdA inhibits Wnt/β-catenin signaling in a dose-dependent manner, which occurs primarily through inactivating the Rho GTPases rather than caspase-dependent β-catenin degradation (Bezerra et al., 2014). In 2020, another *in vivo* study further showed that TcdA inactivates Rac1 by glycosylation, by which the Wnt/β-catenin signaling pathway is inhibited and β-catenin is subsequently prevented from entering the nucleus, thereby inhibiting cell proliferation (Martins et al., 2020). Additionally, it has been reported that TcdA and TcdB affect the Hippo pathway, which is essential for tissue homeostasis and regeneration. YAP and TAZ, the downstream transcriptional co-activators of the Hippo pathway, are able to promote cell proliferation and intestinal regeneration (Gregorieff et al., 2015). The toxins have been demonstrated to inhibit YAP and TAZ by inactivating GTPases in IECs (Song et al., 2021). These findings suggest that these pathways may serve as therapeutic targets for CDI. Numerous studies have shown that TcdA and TcdB induce apoptosis in IECs by glycosylating Rho GTPases, which depends on the activation of caspase-3 (Qa’Dan et al., 2002; Nottrott et al., 2007). Caspase-6, -8, and -9 are also involved in toxins-induced apoptosis (Brito et al., 2002; Carneiro et al., 2006). TcdB has been found to induce apoptosis through both caspase-dependent and caspase-independent pathways. Caspase-dependent apoptosis involves the activation of caspase-3, while caspase-independent apoptosis may result from the GTD-induced inactivation of Rho, Rac, and Cdc42 (Qa’Dan et al., 2002). TcdA and TcdB can activate caspase-dependent apoptosis through a death receptor or mitochondria-dependent pathway (Elmore, 2007). In the mitochondria-dependent pathway, toxins can release cytochrome c and activate caspase-9 by altering mitochondrial outer membrane permeability, and this process is regulated by the anti-apoptotic members of the Bcl-2 family (Matarrese et al., 2007; Matte et al., 2009). Meanwhile, in the death receptor pathway, caspase-8 is activated by transmembrane death receptors, such as TNF-α, Fas, or IFN-γ, subsequently triggering IECs apoptosis (Gerhard et al., 2008). Recently, several molecules, such as the globular heads of C1q and junction plakoglobin, have been revealed *in vitro* and *in vivo* to play a crucial role in toxin-induced apoptosis of IECs in a mitochondria-dependent manner (Liang et al., 2020; Li et al., 2022). Moreover, it has been identified that the activation of caspase-3/7 by the intrinsic apoptotic pathway is crucial in triggering the apoptosis of IECs *in vivo*, and this activation does not rely on the pyrin inflammasome (**Figure 3A**) (Saavedra et al., 2018). Enteric glial cells (EGCs) are components of the enteric nervous system and contribute to maintaining normal intestinal function and the integrity of the intestinal barrier. Studies have indicated that TcdB can induce apoptosis in EGCs through a caspase-dependent but mitochondria-independent pathway which is not influenced by Bcl-2 family members (Macchioni et al., 2017; Fettucciari et al., 2017). Furthermore, stimulating EGCs with pro−inflammatory cytokines significantly enhanced the TcdB-induced apoptosis (Fettucciari et al., 2022). Recent studies have also proposed that adenosine receptors A2A and A2B, and the calcium-permeable channel TRPV4 can participate in modulating toxin-mediated apoptosis of EGCs and inflammatory responses in mice with CDI (Costa et al., 2022; Pacifico et al., 2025). Unlike IECs, myeloid cells like macrophages and dendritic cells show high expression of the cytosolic receptor pyrin (Sharma et al., 2018). In response to the GT activity of TcdA and TcdB, pyrin forms an inflammasome complex which functions as a sensor to activate inflammatory caspases, such as caspase-1 (Xu et al., 2014). Caspase-1 can initiate the maturation and the release of IL-1β and IL-18, and trigger an inflammatory programmed cell death known as pyroptosis (Bergsbaken et al., 2009; Jamilloux et al., 2018). Recent studies have proposed that *C. difficile* toxins can inactivate RhoA GTPases through the glycosylation of GTD, and subsequently activate the pyrin inflammasome to induce pyroptosis in mouse macrophages and human peripheral blood mononuclear cells (Xu et al., 2014; Gao et al., 2016; Van Gorp et al., 2016). Autophagy, as a pro-death mechanism under certain conditions, mediates degradation of cellular components via the lysosomal system (Kloft et al., 2010). Numerous studies have shown that autophagy plays a significant role in the pathogenicity of microorganisms (Wang et al., 2025b). In various autophagy-deficient cell lines, researchers have demonstrated that TcdB promotes the formation of the phosphoinositide 3-kinase complex and inhibits the mTOR signaling pathway through its GT activity. This, in turn, triggers autophagy and subsequently inhibits host cell proliferation (He et al., 2017). Interestingly, another study has demonstrated that non-toxigenic strains can also induce autophagy in Caco-2 cells, indicating that *C. difficile* induces autophagy through both toxin-dependent and toxin-independent mechanisms (Azimirad et al., 2023). Surface layer protein A of *C. difficile* has been shown to induce autophagy in human IECs, suggesting a potential role for other *C. difficile* virulence factors in regulating the autophagy process (**Figure 3A**) (Amirkamali et al., 2022).

The GT activity is essential for toxin-induced cell death. However, researchers have recently put forward a standpoint claiming that TcdB plays a more significant role in CDI due to its GT-dependent and GT-independent dual effects (Peritore-Galve et al., 2022). An acute intestinal infection mouse model established by GTD-deficient TcdB demonstrated that the GT activity of TcdB was essential for inducing disease symptoms (Yang et al., 2015b). Another mouse infection model established by GTD-deficient *C. difficile* showed that no significant alternation in the load of *C. difficile*, but a complete loss of pathogenicity to the mice was observed, which further confirmed the GT-dependent mechanism of TcdB (Bilverstone et al., 2020). However, under the condition of high concentrations of TcdB, GT-independent effects such as necrosis and pyknosis have been reported, suggesting that the GT-independent action may be concentration-dependent (Chumbler et al., 2012; Wohlan et al., 2014). TcdB-induced GT-independent necrosis depends on the assembly of the NADPH oxidase complex (NOX) in host epithelial cells and the production of reactive oxygen species (ROS) (Farrow et al., 2013). ROS generated by NOX is regulated by Rac1 (Hordijk, 2006). The GTD of TcdB rapidly accumulates to reach sufficient levels and drives early cell death in a Rac1-dependent manner. The loss of functional Rac1 can inhibit the early cell death induced by high concentrations of TcdB (Beer et al., 2018). Furthermore, TcdB can induce a Ras-dependent cell death termed pyknosis, in which Ras serves as a central upstream regulator of the GT-independent effect of TcdB (**Figure 3A**) (Stieglitz et al., 2022). The mechanism and significance of the cytotoxicity of *C. difficile* toxins remain to be further clarified.

### 3.4 Toxin-mediated host immune response

Multiple studies have shown that TcdA and TcdB cause tissue damage not only by direct cytotoxicity, but also by inducing inflammatory response. TcdA and TcdB can stimulate immune cells such as monocytes and macrophages, and trigger the secretion of inflammatory mediators, including IFNγ, TNF-α, IL-1β, IL-6, IL-8, IL-23, MIP-1α, and MIP-2 (Lee et al., 2007; Hasegawa et al., 2012; McDermott et al., 2017; Castagliuolo et al., 1998; Ishida et al., 2004). Neutrophils and other inflammatory cells were recruited by these inflammatory mediators to amplify the inflammatory cascade, promote vascular hyperpermeability and interstitial edema, and exacerbate intestinal tissue injury (**Figure 3B**) (Morteau et al., 2002; Madan and Petri, 2012; Saleh et al., 2019).

An early study has reported that TcdA interacted with specific surface receptors on rabbit neutrophils to activate a G protein-dependent signaling pathway, which induced neutrophil migration and tissue damage (Kelly et al., 1994a). Furthermore, TcdA has been found to induce monocyte necrosis, IL-1β release, and IL-8 production through the activation of ERK and p38 MAP kinase signaling pathways. It was also suggested that the activation of MAP kinase may not be related to the glycosylation of Rho proteins (Warny et al., 2000). Meanwhile, TcdA can also stimulate the upregulation of IL-8 and monocyte chemotactic protein 1 by activating the NF-κB signaling pathway, thereby inducing inflammatory responses in the intestinal mucosa (Kim et al., 2006). Another study has suggested that the endocytosis pathway of TcdA is necessary for the induction of TNF-α, and TcdA-induced secretion of TNF-α is dependent on its GT activity (Sun et al., 2009). Toll-like receptors (TLRs) are the primary components of the immune system, playing a crucial role in detecting pathogen-associated molecular patterns and in activating both innate and adaptive immune responses (Hsieh et al., 2025). A study revealed the important role of TLR9 in the pathogenesis of TcdA. TcdA binds to bacterial DNA to form a stable complex, which enters cells via a cell-penetrating peptide-like domain, and activates TLR9 pathway to trigger inflammatory responses (Chen et al., 2020a). Meanwhile, another study revealed that the activation of TLR5 signaling played a protective role against CDI, and it was presumed that TLR5 may protect IECs by inducing anti-apoptosis and cell proliferation (Jarchum et al., 2011).

TcdA and TcdB could induce the release of IL-1β by activating the inflammasome, which in turn triggers inflammation and intestinal damage, suggesting that the inhibition of the inflammasome or IL-1β signaling could be potential new strategies for treating CDI (Ng et al., 2010). Further research has indicated that the activation of inflammasomes and IL-1β signaling played a key role during the production of IL-23 stimulated by TcdA and TcdB (Cowardin et al., 2015). IL-23 is a key factor for driving neutrophil recruitment and the innate inflammatory response in *C. difficile* associated colitis, as confirmed in both human colon samples and animal experiments (Buonomo et al., 2013; McDermott et al., 2016). A retrospective study has shown that anti-IL-23 treatments significantly reduce the probability of all-cause death within 30 days, further confirming the inflammatory role of IL-23 in CDI patients (Madden et al., 2025). A transcriptome analysis revealed that although TcdA played a regulatory role in these responses, TcdB was actually the primary factor for inducing host innate immunity and pro-inflammatory responses (Carter et al., 2015). Notably, necrosis induced by TcdB is associated with ROS production mediated by NOX. Inhibition of ROS generation or abrogation of ROS can protect the colon from TcdB-induced damage (Farrow et al., 2013). Additionally, it has been reported that TcdB targets FZD1/2/7 in gut-innervating afferent neurons and CSPG4 in pericytes, releasing neuropeptide substances and inflammatory cytokines, which leads to neurogenic inflammation and subsequently causes CDI-associated histopathology in mouse models (Manion et al., 2023). This finding revealed a novel mechanism of TcdB inducing inflammation and offered a new approach for the targeted therapy of CDI.

However, a recent study suggested that toxin-induced host inflammation may offer potential benefits for *C. difficile* by altering the host’s nutritional environment and the structure of the gut microbiome (Fletcher et al., 2021). Another study has found that IL-33 stimulates the activation of colonic group 2 innate lymphoid cells, which in turn can prevent CDI. While the down-regulation of IL-33 results in severer illness and increased mortality. Moreover, the prevention of *C. difficile* associated mortality and epithelial cell damage by IL-33 is independent of bacterial load or toxin expression (Frisbee et al., 2019). Additionally, researchers have discovered that IL-25 maintained intestinal barrier integrity during CDI by inducing the increase of eosinophilia, and restoring the suppressed expression of IL-25 in CDI could decrease mortality and morbidity (Buonomo et al., 2016). These results indicate that both IL-25 and IL-33 may play protective roles in CDI. Further studies are needed to clarify the relationship between *C. difficile* toxins and host inflammation.

## 4 Strategies for treating and preventing CDI

The American College of Gastroenterology clinical guidelines in 2021 emphasized that vancomycin and fidaxomicin are the first-line treatments for CDI, in combination with parenteral metronidazole for fulminant CDI. Patients experiencing multiple relapses should be treated with FMT (Kelly et al., 2021). That same year, the clinical practice guideline issued by the Infectious Diseases Society of America and the Society for Healthcare Epidemiology of America recommended fidaxomicin over a standard course of vancomycin for patients with either initial or recurrent CDI (Johnson et al., 2021). The European Society of Clinical Microbiology and Infectious Diseases guidelines emphasized the significance of discontinuing predisposing antibiotic therapy and recommended using fidaxomicin for the treatment of CDI when available and feasible (van Prehn et al., 2021). In 2025, updated guidelines from the Australasian Society of Infectious Diseases reaffirm that the cessation of antimicrobial therapies is crucial for CDI prevention and optimal management, while highlighting the significant role of FMT in rCDI (Longhitano et al., 2025).

Treatment for CDI varies worldwide according to these clinical guidelines. Antibiotics remain the first-line treatment option, however they can interfere with the normal gut microbiota composition, leading to the recurrence of infection (Sehgal et al., 2022; Kapandji et al., 2025). At present, several narrow-spectrum antibiotics specifically for *C. difficile*, such as ridinilazole (phase III) and ibezapolstat (phase II), are undergoing clinical trials (Okhuysen et al., 2024; Eubank et al., 2025). Given the remarkable results, these antibiotics are expected to be widely applied clinically in the future. In recent years, it has been recognized that a balanced gut microbiota plays a crucial role in maintaining the health of the host (Liu et al., 2023; Chen et al., 2023). FMT has emerged as a powerful therapeutic approach for managing patients with rCDI. However, the lack of standardization in preparation and administration of fecal material poses inherent risks and limits its large-scale application (Wilcox et al., 2020; Feuerstadt et al., 2022). Although SER-109 and REBYOTA have currently been approved by the FDA to prevent rCDI, more real-world clinical trials are needed to further confirm their safety and efficacy (Gonzales-Luna et al., 2023). Antitoxin-targeted therapies for *C. difficile* toxins, as well as the development of vaccines utilizing inactivated toxins, have also achieved promising results preclinically, and have gradually progressed to clinical trials (Bratkovic et al., 2024). Further studies are still needed to evaluate their long-term efficacy and safety. CDI remains a significant challenge in the health field, and it is urgent to develop new therapies to control the increasing incidence, rising severity and high recurrence rate of CDI. **Table 1** summarizes the existing achievements, limitations, as well as the future direction of progress in CDI treatment.

**Table 1:** Overview of the development of CDI treatment.

Emerging therapeutic strategies	Existing research achievements	Limitations and research gaps	Future research direction	References
Antibiotics	1. The guideline recommends: vancomycin, fidaxomicin, and metronidazole. 2. Narrow-spectrum antibiotics: ridinilazole (phase III), CRS3123 (phase II), and ibezapolstat (phase II).	1. Leading to varying degrees of intestinal flora imbalance. 2. The existing narrow-spectrum antibiotics need additional clinical trials before FDA approval. 3. Antibiotics cannot kill *C. difficile* spores.	1. Conducting additional clinical studies to verify the safety and efficacy of existing narrow-spectrum antibiotics. 2. Searching for ultra-narrow-spectrum agents. 3. Discovering new metabolite adjuvants to promote the transformation of spores into vegetative *C. difficile*.	(Kelly et al., 2021; van Prehn et al., 2021; Okhuysen et al., 2024; Eubank et al., 2025)
Antitoxin-based antibody therapies	1. Monoclonal antibodies: Actoxumab (abandoned), bezlotoxumab (approved), and PA41 (pre-clinical). 2. Polyclonal antibodies: OraCAb (pre-clinical), WPC-40 (clinical trial). 3. Nanobodies: ABA (pre-clinical), ABAB (pre-clinical).	1. Bezlotoxumab was withdrawn from the market on January 31, 2025, leading to a severe shortage of treatment options. 2. Novel antibodies are still under laboratory investigation stage. 3. Antibodies can only effectively neutralize toxins rather than eradicate bacteria.	1. A mixture of monoclonal antibodies showed a higher neutralizing potency, which may be the future research direction. 2. More clinical studies are necessary to explore the safety and effectiveness of the antibodies in the real world. 3. Screening the toxin antibodies by phage display technique. 4. Developing the antibody-drug conjugates.	(Kroh et al., 2018; Roberts et al., 2020; Chen et al., 2020b; Heidebrecht et al., 2021; Feuerstadt et al., 2025a)
Vaccinations	1. Toxoid vaccine: PF06425090 (phase III), a second toxoid vaccine (phase III). 2. Toxoid vaccine with novel adjuvant: GLA 3M-052 LS. 3. RNA-based vaccines: mRNA-lipid nanoparticle vaccine. 4. Protein-based vaccines: surface proteins, intracellular proteins, or polysaccharides.	1. The trial NCT01887912 was terminated because it failed to prevent CDI. 2. Toxoid vaccines can shorten the duration of the disease and lessen its severity, but not eliminate the initial infection or prevent its spread. 3. Poorly effective in immunocompromised patients. 4. At present, there are no available vaccines for CDI on the market.	1. Focusing on the safety and long-term effectiveness of vaccines in clinical trials. 2. Multiple approaches are utilized in the screening of new candidate vaccine targets: based on protein immunoreactivity, adhesive properties, a bioinformatic approach (reverse vaccinology), or a combined method. 3. Developing next-generation vaccine adjuvants.	(de Bruyn et al., 2021; Razim et al., 2023; Alameh et al., 2024; Donskey et al., 2024; Naz et al., 2025)
Gut microbiota restoration	1. FMT treatment for rCDI has been recommended by clinical guidelines. 2. Live biotherapeutic products: CP101 (phase II), RBX2660/Rebyota (PUNCH CD3-OLS), SER109 (phase III).	1. Lacking standardization in the preparation and administration of fecal material. 2. The long-term colonization dynamics after FMT and effective biomarkers to predict treatment responses remain unclear. 3. It has no antibacterial activity and should be used after antibiotic treatment.	1. Standardizing the administration and conducting more real-world clinical trials to verify the safety and efficacy. 2. Tracking the dynamic changes of the microbiota after FMT by 16S or metagenomic analysis. 3. Identifying microbial markers through the integration of multi-omics analysis, including the combination of metabolomics with 16S or metagenomic sequencing.	(Sims et al., 2023; Allegretti et al., 2025a; Feuerstadt et al., 2025b; Allegretti et al., 2025b)

### 4.1 Antitoxin-based antibody therapies

The standard therapy for CDI primarily relies on antibiotics, yet it has a high recurrence rate. In recent years, researchers have sought to develop new strategies that target toxins instead of pathogens to reduce recurrence rates. Hence, antitoxin-based antibody therapies have attracted significant attention.

It is confirmed that passive immunization using antitoxin mAbs against TcdA and TcdB can achieve reductions in mortality, morbidity, and recurrence rates to different extents in various animal infection models (Kink and Williams, 1998; Giannasca et al., 1999; Babcock et al., 2006; Yang et al., 2015a). In 2012, novel antitoxin mAbs, anti-TcdA PA50 and anti-TcdB PA41, were successfully generated and humanized. The combination of PA50 and PA41 significantly improved the survival rates in a hamster model of CDI (Marozsan et al., 2012). Subsequently, the mechanism of PA41 was elucidated through a combination of structural, biochemical, and cellular functional studies. PA41 recognizes a highly conserved epitope on the GTD of TcdB and prevents its translocation into the cytosol (Kroh et al., 2018). In addition to studies on animal models, data from CDI patients also supported the feasibility of adopting TcdA and TcdB antibodies. Morever, the serum levels of anti-TcdA and anti-TcdB mAbs were correlated with CDI relapse (Kyne et al., 2001). In a phase II clinical study (NCT00350298), simultaneous administration of CDA1 and CDB1, human mAbs targeting TcdA and TcdB, during antibiotic treatment significantly reduced the recurrence rate of CDI (Lowy et al., 2010; Gupta et al., 2016). Actoxumab and bezlotoxumab are human mAbs against *C. difficile* TcdA and TcdB, respectively, which have been proven effective in several preclinical studies (Gupta et al., 2016). Two phase III clinical trials (NCT01241552 and NCT01513239) also confirmed that bezlotoxumab significantly reduced the recurrence rate and exhibited a safety profile similar to that of the placebo. However, actoxumab failed to exhibit such effect, and the therapeutic effect was not significantly enhanced when combining with bezlotoxumab (Wilcox et al., 2017). Both actoxumab and bezlotoxumab bind to the CROP domains to prevent the toxins from binding to mammalian cells. A study has shown that bezlotoxumab binds to two homologous but distinct epitopes on the CROP domain, thereby preventing TcdB from binding to the surface of host cells (Orth et al., 2014). However, actoxumab cannot bind to both epitopes simultaneously because they are situated on the opposite sides of the CROP domain of TcdA, which may be one reason for its insufficient efficacy (Hernandez et al., 2017). Therefore, exploring anti-TcdA mAbs that target different epitopes may offer improved protection against TcdA. The binding of bezlotoxumab can induce an allosteric change in TcdB, thereby disrupting the CSPG4-binding site (Chen et al., 2021). However, bezlotoxumab does not affect the interaction between TcdB and FZD1/2/7 or Nectin-3, since TcdB binding to these receptors is independent of the CROP domain. This indicates that neutralizing antibodies targeting other toxin domains provide comparable or enhanced protective effects (Manse and Baldwin, 2015). Several animal studies have confirmed that a mixture of mAbs targeting different toxin domains exhibits a higher neutralizing efficacy compared to a single mAb (Davies et al., 2013; Qiu et al., 2016), highlighting the potential value of hybrid mAbs in future research. As the direct neutralizing effect of antibodies is crucial in inactivating toxins, antibody fragments, such as nanobodies, are expected to serve as effective alternatives to full-length mAbs. Researchers have developed a tetravalent, bispecific antibody comprising two V_H_H binding domains against both TcdA and TcdB, namely ABA. It exhibited the ability to simultaneously neutralize TcdA and TcdB and the efficacy in preventing and treating CDI in mice (Yang et al., 2014). Subsequently, ABAB, a tetra-specific antibody consisting of four distinctive toxin-neutralizing V_H_Hs, has shown a broad neutralizing capacity in mice and hamsters (Chen et al., 2020b). Several studies have indicated that although the neutralization of both toxins was necessary for the maximum protection of rodents, the neutralization of TcdB alone seemed to be adequate for mammals, which suggested that the neutralization effect may depend on host species (Leav et al., 2010; Steele et al., 2013).

Currently, bezlotoxumab remains the only anti-toxin antibody approved by FDA to prevent rCDI (Mullard, 2016; Wilcox et al., 2017). However, since Merck, the manufacturer, has announced that bezlotoxumab will be withdrawn from the market on January 31, 2025, many medical institutions will face a severe shortage of treatment options (Feuerstadt et al., 2025a). Additionally, due to the presence of multiple TcdB variants, current antibodies exhibit low neutralizing potency against the TcdB variants of various epidemic pathogenic strains. Recently, several new technologies, such as phage display technique, have emerged to facilitate the screening of new *C. difficile* toxin antibodies (Kordus et al., 2023; Raeisi et al., 2023). However, there is still a gap between laboratory achievements and clinical efficacy, and further research on *C. difficile* toxin antibodies is necessary. It is important to recognize that the antibodies mentioned above are effective in neutralizing toxins rather than eradicating the bacteria. Therefore, the development of antibody-drug conjugates is anticipated to be a novel research frontier (Wang et al., 2025a). Existing antibody therapies have been discontinued, and the development of new antibodies remains in preclinical studies. Despite the promising results, there are inherent limitations due to the lengthy clinical trial process.

### 4.2 Vaccination

CDI continues to be a significant and costly medical issue, primary prevention is greatly needed. The development of vaccines has prospective efficacy in protecting individuals with high risks of developing CDI. Although there is yet no available vaccine for *C. difficile* on the market, data from several clinical trials have indicated its potential feasibility. *C. difficile* vaccines primarily include inactivated toxins, recombinant toxins, and RNA vaccines, which are designed to induce systemic antibody responses against TcdA and TcdB (Knisely et al., 2016; Tang et al., 2025). A phase II clinical trial (NCT02561195) has demonstrated satisfactory tolerance, bio-safety as well as immunogenicity of the *C. difficile* vaccine in healthy US adults, which supported the further development of the vaccine (Kitchin et al., 2020). It further showed that immune responses to the *C. difficile* vaccine persisted for 48 months after the third dose, and a four-dose administration was found to prolong the immunogenicity up to 3 years with safety in an extension study involving adults aged 65 to 85 years (Remich et al., 2024). However, a phase III clinical trial (NCT01887912) demonstrated that although a bivalent *C. difficile* toxoid vaccine exhibited good immunogenicity and safety, it was ineffective in preventing CDI. Consequently, the study was ultimately terminated (de Bruyn et al., 2021). Another clinical trial (NCT03090191) evaluated PF-06425090, a detoxified toxin-A/B vaccine for primary CDI prevention. It demonstrated that while the vaccine significantly reduced the CDI events requiring treatment and effectively shortened the symptom duration, it also failed to lower the incidence of primary CDI events (Donskey et al., 2024). Various clinical studies have shown that toxoid vaccines can shorten the duration of the disease and lessen its severity, but do not eliminate the initial infection or prevent its spread. The development of next-generation vaccines against *C. difficile* faces numerous challenges.

Given the suboptimal performance of aluminum as an adjuvant in *C. difficile* vaccines for inducing immunity, researchers have shifted their focus to seeking novel adjuvants. A recent study has revealed that GLA 3M-052 LS, a dual Toll-like receptor ligand liposome, can enhance the immunogenicity of TcdB vaccines in mice (Naz et al., 2025). This breakthrough holds promise for advancing the development of next-generation vaccines against CDI. Recently, mRNA vaccines have shown significant potential in combating a variety of pathogens. Researchers have successfully developed an mRNA-lipid nanoparticle vaccine targeting *C. difficile* toxins and virulence factors, and have verified its effectiveness in preventing CDI and promoting bacterial clearance in multiple clinically relevant animal models (Alameh et al., 2024). Numerous studies have indicated that toxoid vaccines do not prevent the transmission of bacteria among patients or eradicate the pathogen during the initial stages of infection. Therefore, an ideal vaccine should incorporate additional antigenic components that provoke an immune response early in the infection process, such as surface proteins or polysaccharides (Razim et al., 2023). These studies remain at the stage of animal experiments and urgently require further clinical trials to verify their safety and efficacy. Although the development of *C. difficile* vaccines still faces multiple challenges, it is of great significance to provide better solutions for preventing CDI.

### 4.3 Gut microbiota restoration

Recently, FMT has garnered significant attention from researchers due to its potential in preventing rCDI. Studies suggest that microbial imbalance is closely associated with the occurrence of CDI, with the widespread use of antibiotics being one of the main causes of gut microbiota disruption (Surawicz, 2009). Antibiotic treatment-induced dysbiosis leads to the excessive proliferation of harmful bacteria such as *C. difficile*, resulting in subsequent infections (Leffler and Lamont, 2015). Research has shown that the abundance of *Enterococci* significantly increases, while beneficial bacteria such as *Bifidobacterium* and *Ruminococcus* significantly decrease in patients treated with β-lactam antibiotics, leading to high susceptibility to *C. difficile* (Berkell et al., 2021). Furthermore, a study found that patients with CDI have already shown lower microbial diversity before antibiotic treatment, further suggesting that changes in gut microbiota diversity are related to the occurrence and development of CDI (Chen et al., 2022). The prognosis of CDI may be improved by restoring the balance of intestinal microbiota. Recent studies have shown that probiotics such as Firmicutes can effectively inhibit *C. difficile* growth and simultaneously promote the recovery of beneficial intestinal flora in patients with CDI (Herrera et al., 2021). Additionally, results from various animal models have indicated that the microbial therapies can also inhibit bacterial growth and toxin secretion by altering intestinal metabolites, such as short-chain fatty acid, lactic acid, and bile acid (Buffie et al., 2015; Li et al., 2024, Li et al., 2025). Recently, caffeic acid phenethyl ester and equol have been identified to reduce intestinal damage from toxins by screening the natural compounds library. They inhibit bacterial growth and toxin secretion by regulating intestinal metabolism in mouse models while ensuring the integrity of the intestinal microbiota, revealing their potential as a therapeutic for the management of CDI (Guo et al., 2025a, Guo et al., 2025b). A healthy gut microbiota not only inhibits the growth of *C. difficile* but also activates the host’s immune response. A restored microbiota can regulate immune cells and promote the release of anti-inflammatory factors, thereby reducing infection-induced inflammatory responses (Chiu et al., 2021; Yang et al., 2024). Microbial metabolites, including succinate and citrulline, have been demonstrated in CDI mouse models to exert anti-inflammatory effects by activating immune cells, effectively protecting against CDI-induced damage (Xie et al., 2025; Kellogg et al., 2025). Therefore, the restoration of gut microbiota as an adjunctive strategy in treating CDI has demonstrated its importance and potential.

FMT is recommended by multiple clinical guidelines for preventing rCDI due to its benefits in restoring the balance of the gut microbiota (van Prehn et al., 2021; Johnson et al., 2021). A clinical trial (NCT03005379) enrolled a veteran population with rCDI to test the efficacy and safety of capsule-delivered FMT, which contains lyophilized microbiota isolated from fecal material from standardized donors. Unfortunately, the trial was terminated because there were no significant advantages of FMT in preventing rCDI or reducing mortality rates (Drekonja et al., 2025). Due to the lack of standardization in the compositions, dosages, and administrations of fecal material, the outcomes of FMT have been less than satisfactory. Recently, several live biotherapeutic products have shown encouraging results. CP101, a full-spectrum oral microbiome therapy, has been proven to be more effective than placebo in reducing the recurrence rate of CDI, with comparable safety profiles, in phase II clinical trials (NCT03110133, NCT03497806) (Allegretti et al., 2025b). The phase III clinical trial of CP101 (NCT05153499) is still in the recruitment stage for subjects (Gonzales-Luna et al., 2023). SER-109 is an oral capsule consisting of live, purified Firmicutes bacterial spores. It is designed to achieve therapeutic goals via a dual mechanism involving competitive metabolism and bile acid regulation. SER-109 has completed phase II (NCT02437487), phase III (NCT03183128) and open-label phase III (NCT03183141) trials (McGovern et al., 2021; Feuerstadt et al., 2022; Sims et al., 2023). The results have consistently shown that SER-109 has good tolerability and significantly reduces CDI recurrence rates. Additionally, the SER-109 group showed increased production of secondary bile acids, which inhibited the germination and growth of *C. difficile* spores (Feuerstadt et al., 2022). REBYOTA (formerly known as RBX2660), a single-dose broad consortia microbiota based live biotherapy, has achieved significant data in phase II and phase III clinical studies (Khanna et al., 2022; Orenstein et al., 2022; Dubberke et al., 2018). Recently, in a PUNCH CD3-OLS clinical trial (NCT03931941), REBYOTA has further demonstrated its safety and efficacy in preventing rCDI (Feuerstadt et al., 2025b). Meanwhile, the results of another PUNCH CD3-OLS clinical trial (NCT03244644) have shown that the efficacy of REBYOTA in preventing rCDI is not weakened in patients with inflammatory bowel disease (Allegretti et al., 2025a). Currently, SER-109 and REBYOTA have been approved by the FDA to prevent rCDI. However, live biotherapeutic products still need to be carried out on the basis of antibiotic treatment. More large-scale, real-world randomized controlled trials are needed to further verify the efficacy and safety of microbial therapy for rCDI. Moreover, exploring optimal intervention timing and methods across different clinical scenarios is necessary to achieve better management of rCDI.

### 4.4 Novel small molecule strategies

Antibiotics are the preferred small molecule agents recommended for the treatment of CDI. However, they often lead to recurrence by interfering with the normal intestinal flora (Leffler and Lamont, 2015). There has been a shift in focus towards small molecule strategies without direct bactericidal or bacteriostatic activity. Currently, screening libraries of medications approved for other diseases is a significant initiative for searching potential treatment agents for CDI.

Niclosamide, an anthelmintic drug, has recently been found to neutralize the cytotoxic effects of TcdA, TcdB, and CDT in infected mice. It functions by inhibiting the process of pore formation while maintaining the balance of the intestinal flora (Tam et al., 2018). Cholesterol in the cell membrane is crucial for the formation of pores by TcdA and TcdB (Giesemann et al., 2006). Statins, a group of cholesterol-lowering medications used in clinics, have been demonstrated to prevent the cytotoxic effects of these toxins *in vitro*, highlighting their potential therapeutic benefits in treating CDI (Papatheodorou et al., 2019). Additionally, the antiarrhythmic drug amiodarone has been confirmed to provide protection against both TcdA and TcdB by inhibiting cholesterol biosynthesis under *in vitro* conditions (Schumacher et al., 2023). Another study has revealed that calcium channel signaling acts as a key mediator of TcdB-induced necrosis through small molecule screening, further suggesting that the calcium channel blocker amiodarone may offer a general protective effect against the severe consequences of CDI (Farrow et al., 2020). Future studies focusing on drugs that inhibit cholesterol synthesis in the cell membrane and other molecules that block toxin action may facilitate the development of effective therapies against CDI. Auranofin, an FDA-approved oral anti-rheumatic drug, may reduce spore and toxin production by inhibiting selenium metabolism in *C. difficile*, or by interfering with its biosynthesis *in vivo*. This suggests that Auranofin has potential as a promising therapeutic option for CDI, especially in reducing disease recurrence and controlling nosocomial infections (Hutton et al., 2020). Misoprostol, an FDA-approved stable prostaglandin E1 analogue, has been demonstrated to protect against *C. difficile*-associated mortality by decreasing the intestinal mucosal permeability in mouse models of CDI. Additionally, it contributes to the recovery of gut microbiota following antibiotic perturbation (Zackular et al., 2019). Bile acid metabolites are crucial for influencing the life cycle of *C. difficile* (Buffie et al., 2015). Obeticholic acid, an antagonist of farnesoid X receptors, has been approved for the treatment of primary biliary cholangitis. It was found to decrease the bacterial load and improve the prognosis of CDI in mice by reducing the synthesis of primary bile acids (Jose et al., 2021). Ketotifen, an anti-allergy drug, was found to inhibit enteritis induced by TcdA in rats, primarily by suppressing the release of mediators derived from mast cells and neutrophils (Pothoulakis et al., 1993). The antidepressant amoxapine, the respiratory stimulant doxapram, and the antipsychotic trifluoperazine were recently found to effectively reduce the bacterial burden and toxin levels in mice with CDI. Moreover, these drugs have minimal impact on the composition of the microbiota as they have neither bacteriostatic nor bactericidal properties (Andersson et al., 2018). The team further explored the mechanism of action using RNA-seq technology and discovered that these drugs protect against CDI by modulating the host innate immune defenses (Andersson et al., 2020). These results emphasize the importance of immune regulation as a potential therapeutic option for CDI. Berberine, a natural compound found in traditional Chinese medicine, exhibits multiple biological functions, including anti-inflammatory and antioxidant properties. It is used clinically to treat intestinal infections. Recent studies have revealed its potential protective effects against rCDI in animal models, indicating the potential role of traditional Chinese medicine in the treatment of CDI (Wang et al., 2025).

Additionally, numerous new therapies are currently under development, including viruses, bacteriophages and their derivatives such as endolysins and tailocins, as well as microbial metabolites like antimicrobial peptides and α-defensins (Jank et al., 2008; Danis-Wlodarczyk et al., 2021; Phothichaisri et al., 2022; Mondal et al., 2020; Ghosh et al., 2024; Lietz et al., 2025). These novel treatments have demonstrated their potential in the preclinical stage. Despite promising results, a lengthy clinical trial process is the basis for the future clinical applications in the treatment and prevention of recurrent and refractory CDI.

## 5 Conclusion

In this review, we summarized the epidemiology of CDI, the action mechanism of TcdA and TcdB, the interaction between toxins and host cells, and the progress of treatments. As one of the most common healthcare-associated diseases, CDI still has many unsolved scientific questions (Di Bella et al., 2024). For instance, the cell surface receptor for toxins warrants further investigation. The role of GTPases as toxin targets in the cytoplasm has been established. However, when the toxin reaches a certain concentration, the GT-independent cell death pathway and the associated pathological damage require further investigation (Orrell and Melnyk, 2021). With the widespread transmission of various subtypes of virulent *C. difficile* globally, researchers have discovered that these strains display diverse mechanisms of toxin action. Research into the structure of toxin subtypes not only aids in addressing issues at the root of pathogenic mechanisms, but also offers robust support for the development of new targeted drugs. In the exploration of therapeutic strategies, small-molecule metabolites show promising potential. These compounds not only inhibit GT activity of toxins but also restore gut microbiota balance. This dual effect offers a new therapeutic strategy for CDI. Although research on anti-toxin antibodies and CDI vaccines is still very scarce at present (Donskey et al., 2024; Naz et al., 2025), progress in novel narrow-spectrum antibiotics and approval of various live biotherapeutic products have significantly advanced the clinical management of CDI (Sims et al., 2023; Okhuysen et al., 2024; Feuerstadt et al., 2025b).

In conclusion, to fully understand the pathogenic mechanism of CDI and develop new anti-CDI drugs, there is still a long way to go. It is hoped that this review will be beneficial to researchers who are seeking a comprehensive acknowledgement of the action mechanisms of *C. difficile* toxins and recent advances in the treatment of CDI.
